# Exponential Arithmetic Based Self-Healing Group Key Distribution Scheme with Backward Secrecy under the Resource-Constrained Wireless Networks

**DOI:** 10.3390/s16050609

**Published:** 2016-04-28

**Authors:** Hua Guo, Yandong Zheng, Xiyong Zhang, Zhoujun Li

**Affiliations:** 1 State Key Laboratory of Software Development Environment, Beihang University, Beijing 100000, China; zy1406423@buaa.edu.cn (Y.Z.); lizj@buaa.edu.cn (Z.L.); 2 State Key Lab of Mathematical Engineering and Advanced Computing, Wuxi 214000, China; xiyong.zhang@hotmail.com

**Keywords:** wireless networks, self-healing group key distribution, exponential arithmetic, backward secrecy, J0101

## Abstract

In resource-constrained wireless networks, resources such as storage space and communication bandwidth are limited. To guarantee secure communication in resource-constrained wireless networks, group keys should be distributed to users. The self-healing group key distribution (SGKD) scheme is a promising cryptographic tool, which can be used to distribute and update the group key for the secure group communication over unreliable wireless networks. Among all known SGKD schemes, exponential arithmetic based SGKD (E-SGKD) schemes reduce the storage overhead to constant, thus is suitable for the the resource-constrained wireless networks. In this paper, we provide a new mechanism to achieve E-SGKD schemes with backward secrecy. We first propose a basic E-SGKD scheme based on a known polynomial-based SGKD, where it has optimal storage overhead while having no backward secrecy. To obtain the backward secrecy and reduce the communication overhead, we introduce a novel approach for message broadcasting and self-healing. Compared with other E-SGKD schemes, our new E-SGKD scheme has the optimal storage overhead, high communication efficiency and satisfactory security. The simulation results in Zigbee-based networks show that the proposed scheme is suitable for the resource-restrained wireless networks. Finally, we show the application of our proposed scheme.

## 1. Introduction

Wireless sensor networks have drawn a lot of attention because they have demonstrated applicability in practical applications, such as emergency rescue operations and military application. In these applications, the security of the wireless sensor networks should be highly regarded. In wireless sensor networks, resources, including storage and communication bandwidth, are constrained since nodes are powered by battery. To guarantee the secure communication in wireless networks, secure group keys should be distributed to nodes for the purpose of encryption and authentication.

As an issue in wireless networks, packet losses are inevitable and have a negative impact on the group key distribution flows. The packets may never arrive for some target nodes. The most direct way is to request retransmission. However, retransmitting data packets consumes additional communication resources, which increases the burden on the group manager (GM) in the large communication group. In addition, nodes could expose their current locations, which might be considered as privacy in some applications. Designing a secure and efficient group key distribution protocol for the resource-constrained wireless networks is a challenging task.

A self-healing mechanism, introduced by Staddon [1], can solve the above problem for the unreliable networks. The core idea of a self-healing mechanism is that, GM adds some redundant messages to the broadcast messages so that the group members have the capability to recover the missing session keys without requiring the GM to retransmit the missing messages. After Staddon’s first self-healing group key distribution (SGKD) scheme, many SGKD schemes were proposed. Up to now, four kinds of SGKD schemes exist: polynomial based SGKD (P-SGKD) schemes [2,3,4,5,6], vector space secret sharing based SGKD schemes [7,8,9,10], bilinear pairings based SGKD schemes [11,12,13] and exponential arithmetic based SGKD (E-SGKD) schemes [1,2,14,15]. Among them, vector space secret sharing based SGKD schemes and the bilinear pairings based SGKD schemes are inefficient compared with the other two in terms of the storage overhead and the communication cost. In addition, some other self-healing mechanisms are proposed such as mutual-healing [16] and full-healing [17].

Related Works. Staddon *et al.* [1] first proposed a P-SGKD scheme, and transformed it to an E-SGKD scheme. Blundo *et al.* [2] attacks construction 1 in [1] and proposed a novel self-healing mechanism. For the purpose of reducing the communication overhead and storage overhead, Liu *et al.* [18] proposed a new SGKD scheme and proposed two constructions which allow the trade-off between the capability of self-healing and the size of broadcast messages. More *et al.* [19] proposed a novel SGKD scheme, using a sliding window to make the communication overhead and the self-healing capability more balanced. Later, some SGKD schemes were designed [20,21].

Liu *et al.* [18] introduced the first revocation polynomial based SGKD (RP-SGKD) scheme with low storage and communication cost. Hong *et al.* [22] simplified the scheme in [18], which has lower communication overhead. After that, to reduce the communication overhead of the known RP-SGKD schemes, some new RP-SGKD schemes combined with hash chains have been proposed [23,24,25,26]. Unfortunately, these RP-SGKD schemes with hash chains are not resistant to the collusion attack. Recently, Chen *et al.* proposed an efficient RP-SGKD schemes [6] which has constant-size storage cost. Unfortunately, Guo *et al.* [27] found that their scheme is insecure. Zou *et al.* [28] proposed the first access polynomial based SGKD (AP-SGKD) scheme. After that, some AP-SGKD schemes are proposed [29,30,31,32,33,34], which can guarantee the group nodes’ identity privacy and reduce the storage overhead to the constant. More recently, Sun *et al.* [35] proposed an AP-SGKD scheme. However, it is easy to find that almost all of the AP-SGKD schemes are insecure [35,36].

Some vector space secret sharing based SGKD schemes [7,8,9,10] are proposed. These schemes are more flexible, since any particular access structure will not be imposed. However, the access structure requires being chosen in advance so that the maximum number of the revoked users is determined. The shortcoming of these SGKD schemes is that the revoked users should be predetermined. Some bilinear pairings based SGKD schemes [11,12,13] are proposed, which can reduce the storage overhead to constant and resist collusion attack of any revoked users. However, the communication overhead is prohibitively large.

The E-SGKD scheme is an extension of the polynomial based SGKD scheme. Rams *et al.* [21] claimed that almost all of the polynomial-based SGKD schemes can be converted to the E-SGKD schemes. Up to now, there are only four published E-SGKD schemes, *i.e.*, Construction 5 in [1] and Scheme 4 in [2], the scheme in [14,15]. Staddon *et al.* proposed the first E-SGKD scheme (see Construction 5 [1]) based on bivariate polynomial and Lagrange Interpolation. Later, Blundo *et al.* simplified Staddon *et al.*’s scheme and proposed an E-SGKD scheme (see Scheme 4 [2]) based on univariate polynomial and Lagrange Interpolation with lower communication overhead. However, both construction 5 [1] and Scheme 4 [2] do not have backward secrecy and the size of the broadcast is too large. Rams *et al.* [21] pointed out that all known E-SGKD schemes can not offer backward secrecy. In order to solve the backward secrecy and reduce the size of the broadcast message, Rams *et al.* [14] proposed an efficient E-SGKD scheme based on Lagrange Interpolation and sliding windows with backward secrecy. Then, Rams *et al.* [15] improved the scheme [14] and proposed an E-SGKD scheme with lower storage overhead. The sliding windows allow the trade-off between the size of the broadcast message and the self-healing capability.

In this paper, we propose a new mechanism to achieve backward secrecy of the E-SGKD scheme. Compared with existing E-SGKD schemes [14,15] with backward secrecy, our proposed scheme has full self-healing properties, that is, user nodes can recover all of the lost session keys. In Schemes [14,15], user nodes can only recover part session keys determined by sliding windows. In addition, the communication overhead in our proposed scheme is low.

Except for the method of Lagrange Interpolation, there is another method to construct E-SGKD schemes. The core idea is that the computational operations of recovering the session key are moved to the exponent. Based on this idea, in this paper, we present a secure E-SGKD scheme with high efficiency. To make the new scheme easily understood, we first present a basic E-SGKD scheme based on Hong *et al.*’s Construction 2 [22]. In the basic construction, to reduce the users’ storage overhead, only one secret polynomial is selected as a secret polynomial. However, if this secret polynomial is repeatedly used, all basic security properties are destroyed. Hence, a random value $v_{j}$ for each session is chosen to update the secret polynomial for each session. Unfortunately, such an E-SGKD scheme still does not have backward secrecy.

Based on the basic construction, we further present the new E-SGKD scheme with backward secrecy, optimal storage and low communication bandwidth using two strategies. The first strategy is used to construct the revocation polynomials in the broadcast messages, thus achieving backward secrecy. More precisely, the group users, whose identities are used to compute the revocation polynomials, are divided into different subgroups according to their joined sessions. The second strategy, dual chains, are used for efficient seal-healing of the lost session keys. As we know, the hash chain is a useful tool to reduce the communication overhead in efficient seal-healing mechanisms. However, we find that the P-SGKD schemes with hash chains can not be converted to the E-SGKD schemes directly, and we will discuss the details later. To minimize the communication overhead, we introduce the dual chains. The first chain is a traditional hash chain, and the second chain is a key chain. Two chains are combined together to help the active group users compute the lost session keys, which reduces the number of the broadcast messages. Note that these two strategies, especially the first one, can be applied to transform other P-SGKD schemes to E-SGKD schemes. The new E-SGKD scheme has the following advantages:

- The new E-SGKD scheme solves the backward secrecy of E-SGKD schemes perfectly, *i.e.*, the proposed scheme can satisfy the backward secrecy, and furthermore can resist the collusion attack. The construction method of this scheme can be applied to convert other P-SGKD schemes to secure E-SGKD schemes.
- The storage overhead of the new schemes is optimal, *i.e.*, one element in $Z_{p}$.
- Thanks to the dual chains, the new E-SGKD scheme minimizes the communication cost, *i.e.*, the number of the broadcast messages is reduced to the number of the sessions in which new group users join in.
- The new E-SGKD scheme is computationally secure, *i.e.*, its security is based on the discrete logarithm problem.

The rest of the paper is arranged as follows. Section 2 defined the security model of this paper. Section 3 presents the basic E-SGKD scheme. Section 4 shows the novel E-SGKD scheme. Section 5 introduces the security analysis and performance comparison. Section 6 presents the practicality in the ZigBee network of the novel E-SGKD scheme. Application to Supervisory Control And Acquisition (SCADA) in smart grid is shown in Section 7. The conclusions are presented in Section 8.

## 2. Security Model

In this section, we introduce the network model, the notations and the hypothesis following Rams *et al.*’s survey [21].

### 2.1. Network Model

The network consists of a user node set $U=\{U_{1},\cdots ,U_{N}\}$ and a single GM. GM has rich resources such as large memory space and unlimited energy resources, and powerful ability including high computational ability. Instead, the resources and ability of user nodes are limited. In the resource-constrained networks, especially, the resources of the user nodes are lower.

GM communicates with the group user nodes under the unreliable channel. Message encryption and authentication by a symmetric group key can guarantee the secure group communication. The network is dynamic, and the user nodes may frequently join and leave the network. The leaving nodes may disclose the group key, thus breaking the security of group communication. Hence, the group key should be changed when there are user nodes joining and leaving the group. In addition, a minimal time interval should be set to change the group key even if the network is changeless. Thus, achieving secure group key distribution is necessary.

### 2.2. General Description of SGKD

In order to achieve secure group communication, group keys need to be changed frequently. Group lifetime is divided into epochs called sessions, where each session has a unique group key. In each session, GM distributes a new session key $K_{j}$ to nodes in $G_{j}$ by broadcasting the key updating messages.

Generally speaking, an SGKD scheme consists of six algorithms.

- **SetUp:** GM constructs personal secret $S_{i}$ for each legitimate group node, and sends it to $U_{i}$ by secure channel. $U_{i}$ can use personal secret $S_{i}$ to recover session keys from broadcast messages.
- **Broadcast:** GM creates message $B_{j}$ from $K_{j}$ according to the following conditions:
  - There exists a algorithm *η*, which for all $i:U_{i}\in G_{j}$, can recover $K_{j}$ with the knowledge of $S_{i}$, that is: $K_{j}=\eta \left(B_{j},S_{i}\right)$.
  - For any set of nodes $R\subset U\backslash G_{j}$, there exists no computational algorithm, ς, which can recover $K_{j}$ with the knowledge of personal secrets of all nodes in *R* that is: $K_{j}=\varsigma \left(B_{j},{\left\{S_{l}\right\}}_{l:U_{l}\in R}\right)$ is not feasible.
- **SessionKeyRecovery:** This algorithm is executed by user nodes. Each member $U_{j}\in G_{j}$ recovers key $K_{j}$ from broadcast message $B_{j}$ with her personal secret $S_{i}$ that is: $K_{j}=\eta \left(B_{j},S_{i}\right)$.
- **SelfHealing:** This algorithm is executed by user nodes to recover lost session keys. Given *l*, *r*, there exists an algorithm *ζ*, which can recover $K_{j}$ with the knowledge of $B_{r}$ by node $U_{i}\in G_{l}\cap G_{j}\cap G_{r}$, that is $K_{j}=\zeta \left(B_{r},S_{i}\right)$, where l<j<r.
- **GroupMemberAddition:** When a node $U_{i}$ joins the group, GM sends his personal secret $S_{i}$ via a secure channel.
- **GroupMemberRevocation:** When $U_{i}$ is revoked from the group. The GM starts a new session and updates the session key, which can not be computed by $U_{i}$.

### 2.3. Definition of Self-Healing Group Key Distribution

In this subsection, we introduce the definition and security properties of SGKD scheme. In order to facilitate the narrative, we first list the notations in Table 1.

**Definition 1.**  *(self-healing key distribution with* mt-*revocation capability). The scheme has* mt-*revocation capability and self-healing property if*

*(1)*  *For a legitimate user* $U_{i},U_{i}\in G_{j}^{j^{\prime }},1\leq j^{\prime }\leq j$, *the session key* $K_{j}$ *can be computed by the j-th broadcast message* $B_{j}$, *and* $U_{i}$*’s personal secret* $S_{i}$.
*(2)*  *Either broadcast packet* $B_{j}$ *or personal secret* $S_{i}$ *alone can obtain any information about* $K_{j}$ (j≥1).
*(3)*  mt-*revocation capability: For all* $U_{i}\notin R_{j}$, $U_{i}$ *can compute* $K_{j}$ *if given the j-th broadcast message* $B_{j}$. *However, the revoked user* $U_{i}\in R_{j}$ *can not, where* $R_{j}=\left\{R_{j}^{1},R_{j}^{2},\cdots ,R_{j}^{j}\right\}$ *and* $R_{j}^{j^{\prime }}$, *denote the users joining the group in session* $j^{\prime }$ *and revoked before and in session j.*
*(4)*  *Self-healing property: For any j (*$1\leq j_{1}\leq j\leq j_{2}$*), a user,* $U_{i}$ *(*$U_{i}\in G_{j_{1}}\cap G_{j_{2}}$*), can recover the session key* $K_{j}$ *from broadcast messages* $B_{j_{2}}$.

**Definition 2.**  *(*mt-*wise forward secrecy). The scheme has* mt-*wise forward secrecy, if all users in* $R_{j}$ *can not obtain information about* $K_{j+1}$ *even knowing session keys* $K_{j^{\prime }}$ *(j’<j), where* $R_{j}\subseteq U$, $|R_{j}|\leq jt$, *and* $R_{j}$ *contains all users revoked before session j.*

**Definition 3.**  *(*any-*wise backward secrecy). The scheme has any-wise backward secrecy if users in* $D_{j}$ *can not obtain information* $K_{j}$ *even knowing session keys* $K_{j^{\prime }}$ *(*$j^{\prime }>j$*), where* $D_{j}$ *denotes users joining the group after session j (*$D_{j}=\left\{D^{j+1},D^{j+2},\cdots \right\}\subseteq U$*) and* $D^{j^{\prime }}$ *contains users joining the group in session* $j^{\prime }$ $\left(j^{\prime }\geq j+1\right)$.

**Definition 4.**  *(resistance to* mt-*wise collusion attack). The scheme has* mt-*wise collusion resistance capability if given any two disjoint sets* $R_{j_{1}}$,$D_{j_{2}}$, *users in* $R_{j_{1}}$ *colluding with users in* $D_{j_{2}}$ *can not recover* $K_{j}\left(j_{1}\leq j\leq j_{2}\right)$ *even knowing* $\{B_{1},B_{2},\cdots ,\left\{S_{i}|U_{i}\in R_{j_{1}}\right\}\}⋃$ $\left\{B_{1},B_{2},\cdots ,\left\{S_{i}|U_{i}\in D_{j_{2}}\right\}\right\}$.

## 3. The Basic E-SGKD Scheme

Rams *et al.* [21] pointed out that almost all of the P-SGKD schemes can be converted to the E-SGKD schemes. Up to now, all P-SGKD schemes are divided into two classes based on if they use Lagrange Interpolation or not. As we surveyed in Section 1, the published E-SGKD schemes, Construction 5 [1], Scheme 4 [2], the schemes [14,15] are constructed based on the P-SGKD schemes with Lagrange Interpolation.

The other kind of P-SGKD schemes, without Lagrange Interpolation, can be divided into another two classes based on if they use hash chains or not. We checked all P-SGKD schemes without Lagrange Interpolation one by one, and found that the P-SGKD schemes with hash chains can not move the computational operations from the polynomial to the exponential, since the recursion of the one-way hash chain could not hold on once transferring the computation to the exponential. Precisely speaking, it is easy to compute H(H(x)) from H(x) while it’s hard to compute $g^{H(H(x))}$ from $g^{H(x)}$. On the other hand, the revocation polynomial based SGKD schemes without hash chains are suitable to be transformed to E-SGKD schemes, such as Scheme 3 in [18] and Scheme 2 in [22]. Since the transformation method is similar, in this paper, we take Hong *et al.*’s Scheme 2 as an example to construct the basic E-SGKD Scheme.

### 3.1. The Basic Construction

The basic construction includes five procedures: SetUp, Broadcast, SessionKeyRecovery, GroupMemberAdddtion and GroupMemberRevocation.

- **SetUp**
  Suppose $G_{1}=\left\{U_{1},U_{2},\cdots ,U_{N}\right\}$ denotes the users who join the group in the initial session. Each user $U_{i}$ has a unique identity *i*. GM randomly selects a *t*-degree polynomial $f\left(x\right)=a_{0}+a_{1}x+\cdots +a_{t}x^{t}\in F_{p}\left[x\right]$ as a secret masking polynomial. Then, the GM distributes the personal secret $S_{i}=\left\{f\left(i\right)\right\}$ to each user $U_{i}\in G_{1}$ via a secure channel, where using secret splitting algorithms in [37] has a better secrecy compared with distributing f(i) to $U_{i}$ directly.
- **Broadcast**
  Suppose $R_{j}=\left\{r_{1}^{j},r_{2}^{j},\cdots ,r_{\omega_{j}}^{j}\right\}$ denotes a set of users who are revoked before and in session *j*, where $|R_{j}|=\omega_{j}\leq t$.
  - The GM constructs the *j*-th revocation polynomial as
    $$r_{j}\left(x\right)=\left(x-r_{1}^{j}\right)\left(x-r_{2}^{j}\right)\cdots \left(x-r_{\omega_{j}}^{j}\right)$$
    where $r_{j^{\prime }}^{j}\;\left(1\leq j^{\prime }\leq \omega_{j}\right)$ denotes the identity of user $U_{j^{\prime }}$
  - The GM selects a random value $K_{j},v_{j}$ from $F_{p}$, and computes $g^{v_{j}}$ and
    $$g^{P_{j}\left(x\right)}=g^{r_{j}\left(x\right)K_{j}+v_{j}\cdot f\left(x\right)}$$
    Then, the GM constructs the broadcast message as
    $$B_{j}=\left(\cup_{i=1}^{j}R_{i}\right)\cup \left\{g^{P_{1}\left(x\right)},g^{P_{2}\left(x\right)},\cdots ,g^{P_{j-1}\left(x\right)},g^{P_{j}\left(x\right)}\right\}\cup {\left\{g^{v_{j^{\prime }}}\right\}}_{j^{\prime }=1,2,\cdots ,j}$$
    Note that if $P_{j}\left(x\right)=b_{0}+b_{1}x+\cdots +b_{t}x^{t}$, $g^{P_{j}\left(x\right)}=g^{b_{0}}\cdot {\left(g^{b_{1}}\right)}^{x}\cdots {\left(g^{b_{t}}\right)}^{x^{t}}$. Let $g^{P_{j}\left(x\right)}$ in $B_{j}$ denote the sequence of $\left\{g^{b_{0}},g^{b_{1}},\cdots ,g^{b_{t}}\right\}$.
- **SessionKeyRecovery**
  - For a legitimate user $U_{i}$, $U_{i}\in G_{j}$ recovers the *j*-th session key $g^{K_{j}}$ by broadcast message $B_{j}$ as follows:
    * $U_{i}$ first uses his personal secret f(i) to compute ${\left(g^{v_{j}}\right)}^{f(i)}$.
    * $U_{i}$ computes $g^{P_{j}\left(i\right)}$.
    * $U_{i}$ evaluates $r_{j}\left(i\right)$.
    * Since
      $$g^{P_{j}\left(i\right)}=g^{r_{j}\left(i\right)K_{j}+v_{j}f\left(i\right)}$$
      $U_{i}$ computes the session key as
      $$g^{K_{j}}={\left(\frac{g^{P_{j}\left(i\right)}}{g^{v_{j}f\left(i\right)}}\right)}^{\frac{1}{r_{j}\left(i\right)}}$$
  - Similarly, $U_{i}$ can recover the lost session keys $g^{K_{j^{\prime }}}$ by using $R_{j^{\prime }}$, $g^{P_{j^{\prime }}\left(x\right)}$, $g^{v_{j^{\prime }}}$ ($1\leq j^{\prime }<j$) adopting the same method, *i.e.*, self-healing property.
  - For a revoked user $U_{i}\in R_{j}$, $r_{j}\left(i\right)=0$. Thus, he can not obtain information about the session key $g^{K_{j}}$.
- **GroupMemberAddition**
  When a user, $U_{k}$, joins the group in session *j*, the GM randomly selects a unique identity $k\in F_{p}$ at random, and $U_{k}$ gets his personal secret $S_{k}=\left\{f\left(k\right)\right\}$ from the GM via a secure communication channel. For security, GM starts a new session.
- **GroupMemberRevocation**
  When a user, $U_{i}$, is revoked in session *j*, the GM then includes (x−i) in $r_{j}\left(x\right)$ and starts a new session.

**Remark 1.**  *In order to guarantee that the users’ personal secret can be reused, we choose a mask value* $v_{j}$ *in session j to multiply the secret polynomial. Thus, different sessions have different secret values, which contributes to the constant storage overhead.*

### 3.2. The Security Problem

It is easy to analyze that the above basic E-SGKD scheme satisfies the forward security and has *t*-revocation capability. Unfortunately, it has an obvious weakness, *i.e.*, it can not achieve the backward secrecy. More precisely, for a user $U_{i}$ who joins the group in session *j*+1, he can recover the session key $g^{K_{j}}$ by the *j*-th broadcast message $B_{j}$ as follows:

- $U_{i}$ computes $g^{P_{j}\left(i\right)}$.
- Since $U_{i}$ is not a revoked user in session *j*, *i.e.*, $i\notin R_{j}$, $r_{j}\left(i\right)\neq 0$. Thus, $U_{i}$ computes $r_{j}\left(i\right)$.
- Note
  $$g^{P_{j}\left(i\right)}=g^{r_{j}\left(i\right)K_{j}+v_{j}f\left(i\right)}$$
  $U_{i}$ computes the *j*-th session’s session key $g^{K_{j}}$ as
  $$g^{K_{j}}={\left(g^{P_{j}\left(i\right)}/{\left(g^{v_{j}}\right)}^{f(i)}\right)}^{\frac{1}{r_{j}\left(i\right)}}$$

Hence, a new user $U_{i}$, who joins the group in session *j* + 1, recovers the session key $g^{K_{j}}$, even if he is not a legitimate user in the session *j*. Thus, the basic E-SGKD scheme does not satisfy backward secrecy.

### 3.3. The Countermeasure

The reason why the basic E-SGKD scheme can not satisfy the backward secrecy lies in the fact that a user’s personal secret S(i) does not change in the different sessions. As we mentioned above, E-SGKD schemes reduce the size of the personal secret to a constant, *i.e.*, a personal secret S(i) only relates to a user’s identity, no matter when he joins the group. This means a new user, who joins the group later, can use his personal secret to recover the past session keys.

From the above analysis, we know that to achieve the backward secrecy, a user’s personal secret should be changed in the different sessions. However, allocating different personal secrets to different sessions would create linear storage overhead. How to balance the storage overhead and security is a challenging task.

To solve this problem, we consider binding each user’s personal secret with a changed value. More precisely, users are divided into different subgroups according to the sessions in which they join the group. The core idea is described as follows: a unique session identifier $\varepsilon_{j}$ is assigned to each session, and is multiplied with the secret polynomial to produce a new secret polynomial. Thus the personal secret for a user $U_{i}$ who joins the group in session *j* is $\varepsilon_{j}\cdot f\left(i\right)$. As a result, user $U_{i}$ can not recover the previous session keys with his personal secret $\varepsilon_{j}\cdot f\left(i\right)$, *i.e.*, backward secrecy is achieved. On the other hand, it is easy to see that the storage overhead is optimal since $\varepsilon_{j}\cdot f\left(i\right)$ is a random value in $F_{p}$.

The above idea, binding each user’s personal secret with a changed value, can help the scheme gain the backward secrecy. However, this idea can not be directly applied to the basic construction, since the self-healing property is destroyed. To gain efficient self-healing, dual chains are introduced in the basic construction. The first chain is a hash chain, and the second chain is a key chain.

## 4. The Novel E-SGKD Scheme

Motivated by the above idea, in this section, we present a new E-SGKD scheme which consists of five procedures, *i.e.*, SetUp, Broadcast, SessionKeyRecovery, GroupMemberAdddtion, GroupMemberRevocation.

- **Setup**
  GM picks a *t*-degree polynomial $f\left(x\right)=a_{0}+a_{1}x+\cdots +a_{t}x^{t}\in F_{p}\left[x\right]$ at random and keeps it secret. The GM randomly selects a one-way hash function $h_{2}\left(\cdot \right)$: ${\left\{0,1\right\}}^{512}\to {\left\{0,1\right\}}^{128}$. Then, the GM selects a session identifier $\varepsilon_{1}\in F_{p}$ at random. Note that the GM will randomly select a session identifier $\varepsilon_{j}\in F_{p}$ in session *j*. Each user $U_{i}\in G_{1}$ obtains his personal secret $S_{i}=\{\varepsilon_{1}\cdot f\left(i\right)$} through a secure channel, where $G_{1}$ includes the group members who join the group in the initial session and, using secret splitting algorithms in [37], has a better secrecy compared with distributing $\varepsilon_{1}\cdot f\left(i\right)$ to $U_{i}$ directly.
- **Broadcast**
  Suppose $R_{j}=\left\{R_{j}^{1},R_{j}^{2},\cdots ,R_{j}^{j}\right\}$ and $R_{j}^{j^{\prime }}=\left\{U_{r_{1}^{j^{\prime }}},U_{r_{2}^{j^{\prime }}},\cdots ,U_{r_{\omega_{j^{\prime }}}^{j^{\prime }}}\right\}$, where $R_{j}^{j^{\prime }}$ consists of the users joining the group in session $j^{\prime }$ and is revoked before and in session *j*, and $|R_{j}^{j^{\prime }}|=\omega_{j^{\prime }}$ for each $j^{\prime }\left(1\leq j^{\prime }\leq j\right)$. Let $r_{1}^{j^{\prime }},r_{2}^{j^{\prime }},\cdots ,r_{\omega_{j^{\prime }}}^{j^{\prime }}$ denote the revoked users’ identities in $R_{j}^{j^{\prime }}$. Note that $R_{j}^{j^{\prime }}=\emptyset$ if no users leave the group in session $j^{\prime }$.
  - The GM randomly selects $k_{j}^{1}\in F_{p}$, $r_{0}\in F_{p}$, and a one-way hash function $h_{1}\left(\cdot \right)$: ${\left\{0,1\right\}}^{128}\to {\left\{0,1\right\}}^{128}$. The GM uses $r_{0}$ as a seed and $h_{1}\left(\cdot \right)$ as a hash function to construct a hash chain as follows:
    $$\begin{array}{ccc} r_{1} & = & h_{1}\left(r_{0}\right)\\ r_{2} & = & h_{1}\left(r_{1}\right)=h_{1}\left(h_{1}\left(r_{0}\right)\right)=h_{1}^{2}\left(r_{0}\right)\\ & \cdots , & \\ r_{j-1} & = & h_{1}\left(r_{j-2}\right)=\cdots =h_{1}^{j-1}\left(r_{0}\right) \end{array}$$
    Then, the GM constructs the *j*-th keys chain as follows:
    $$\begin{array}{ccc} k_{j}^{2} & = & k_{j}^{1}\cdot r_{1}\\ k_{j}^{3} & = & \left(k_{j}^{2}\right)\cdot r_{2}\\ & \cdots , & \\ k_{j}^{j} & = & \left(k_{j}^{j-1}\right)\cdot r_{j-1} \end{array}$$
  - The GM selects the random value $r_{k}^{\prime j^{\prime }}\in F_{p}\left(1\leq k\leq t-\omega_{j^{\prime }}\right)$ for $R_{j}^{\prime j^{\prime }}$, where $R_{j}^{\prime }=\left\{R_{j}^{\prime 1},R_{j}^{\prime 2},\cdots ,R_{j}^{\prime j}\right\}$ and $R_{j}^{\prime j^{\prime }}=\left\{r_{1}^{\prime j^{\prime }},r_{2}^{\prime j^{\prime }},\cdots ,r_{t-\omega_{j^{\prime }}}^{\prime j^{\prime }}\right\}$. $r_{k}^{\prime j^{\prime }}\left(1\leq k\leq t-\omega_{j^{\prime }}\right)$ are different from each other and are never used for users’ identities. Then, the revocation polynomials are constructed as:
    $$A_{j}^{j^{\prime }}\left(x\right)=\Pi_{i=1}^{|R_{j}^{j^{\prime }}|}\left(x-r_{i}^{j^{\prime }}\right)\Pi_{i=1}^{|R_{j}^{\prime j^{\prime }}|}\left(x-r_{i}^{\prime j^{\prime }}\right),j^{\prime }=1,2,\cdots ,j$$
    where $|R_{j}^{\prime j^{\prime }}\left|=t-\right|R_{j}^{j^{\prime }}|$.
    Note that $R_{j}^{j^{\prime }}$ denotes the set of users joining the group in session $j^{\prime }$ and is revoked before and in session *j*. The number of users in $R_{j}^{j^{\prime }}$ is less than *t*, thus the degree of $\Pi_{i=1}^{|R_{j}^{j^{\prime }}|}\left(x-r_{i}^{j^{\prime }}\right)$ is less than *t*. However, the degree of f(x) is *t*. In the following computation, $A_{j}^{j^{\prime }}\left(x\right)\cdot k_{j}^{j^{\prime }}+v_{j}\cdot \varepsilon_{j^{\prime }}\cdot f\left(x\right)$ may expose some coefficients of $\varepsilon_{j^{\prime }}\cdot f\left(x\right)$. In order to keep the coefficients the polynomial $\varepsilon_{j}f\left(x\right)$ secret, the set $R_{j}^{\prime j^{\prime }}=\left\{r_{1}^{\prime j^{\prime }},r_{2}^{\prime j^{\prime }},\cdots ,r_{t-\omega_{j^{\prime }}}^{\prime j^{\prime }}\right\}$ is randomly selected to pad the revocation polynomials to be *t*-degree, where $r_{k}^{\prime j^{\prime }}\in F_{p}\left(1\leq k\leq t-\omega_{j^{\prime }}\right)$ for $R_{j}^{\prime j^{\prime }}$ is different from each other and never used for users’ identities. Thus, the coefficients of the secret polynomial $\varepsilon_{j^{\prime }}f\left(x\right)$ can be protected better.
  - The GM randomly selects a random value $v_{j}\in F_{p}$. Then, the GM computes
    $$g^{\Phi_{j}^{j^{\prime }}\left(x\right)}=g^{A_{j}^{j^{\prime }}\left(x\right)\cdot k_{j}^{j^{\prime }}+v_{j}\cdot \varepsilon_{j^{\prime }}\cdot f\left(x\right)},\;j^{\prime }=1,2,\cdots ,j$$
    and constructs the broadcast message as
    $$B_{j}=R_{j}\cup R_{j}^{\prime }\cup {\left\{g^{\Phi_{j}^{j^{\prime }}\left(x\right)}\right\}}_{j^{\prime }=1,2,\cdots ,j}\cup {\left\{E_{h_{2}\left(g^{k_{j}^{j^{\prime }}}\right)}\left(r_{j^{\prime }}\right)\right\}}_{j^{\prime }=1,2,\cdots ,j-1}\cup {\left\{E_{h_{2}\left(g^{k_{j}^{j^{\prime }}}\right)}\left(K_{j^{\prime }}\right)\right\}}_{j^{\prime }=1,2,\cdots ,j}\cup g^{v_{j}}$$
    Note that if $\Phi_{j}^{j^{\prime }}\left(x\right)=b_{0}+b_{1}x+\cdots +b_{t}x^{t}$, $g^{\Phi_{j}^{j^{\prime }}\left(x\right)}=g^{b_{0}}\cdot {\left(g^{b_{1}}\right)}^{x}\cdots {\left(g^{b_{t}}\right)}^{x^{t}}$. Let $g^{\Phi_{j}^{j^{\prime }}\left(x\right)}$ in $B_{j}$ be the sequence of $\left\{g^{b_{0}},g^{b_{1}},\cdots ,g^{b_{t}}\right\}$.
- **SessionKeyRecovery**
  - For a legitimate user $U_{i}$, $U_{i}\in G_{j}^{j^{\prime }}$ uses the *j*-th broadcast message to compute the current session key $K_{j}$ and recover the lost session keys as:
    * $U_{i}$ computes $g^{\Phi_{j}^{j^{\prime }}\left(i\right)}$, where
      $$g^{\Phi_{j}^{j^{\prime }}\left(i\right)}=g^{A_{j}^{j^{\prime }}\left(i\right)\cdot k_{j}^{j^{\prime }}+v_{j}\cdot \varepsilon_{j^{\prime }}\cdot f\left(i\right)}$$
      $U_{i}$ computes $A_{j}^{j^{\prime }}{\left(i\right)}^{-1}$, and uses his personal secret $\varepsilon_{j^{\prime }}\cdot f\left(i\right)$ to compute
      $$g^{k_{j}^{j^{\prime }}}={\left(\frac{g^{\Phi_{j}^{j^{\prime }}\left(i\right)}}{g^{v_{j}\cdot \varepsilon_{j^{\prime }}\cdot f\left(i\right)}}\right)}^{\frac{1}{A_{j}^{j^{\prime }}\left(i\right)}}$$
    * $U_{i}$ computes $h_{2}\left(g^{k_{j}^{j^{\prime }}}\right)$ and evaluates $r_{j^{\prime }}$ by decrypting $E_{h_{2}\left(g^{k_{j}^{j^{\prime }}}\right)}\left(r_{j^{\prime }}\right)$. Then, $U_{i}$ obtains $\left\{r_{j^{\prime \prime }}\right\}\left(j^{\prime }\leq j^{\prime \prime }\leq j-1\right)$ by using $h_{1}\left(\cdot \right)$.
    * $U_{i}$ computes $g^{k_{j}^{j^{\prime \prime }}}\left(j^{\prime }\leq j^{\prime \prime }\leq j\right)$ as
      $$\begin{array}{c} g^{k_{j}^{j^{\prime }+1}}={\left(g^{k_{j}^{j^{\prime }}}\right)}^{r_{j^{\prime }}}\\ g^{k_{j}^{j^{\prime }+2}}={\left(g^{k_{j}^{j^{\prime }}}\right)}^{r_{j^{\prime }}r_{j^{\prime }+1}}\\ \cdots \\ g^{k_{j}^{j}}={\left(g^{k_{j}^{j^{\prime }}}\right)}^{r_{j^{\prime }}\cdots r_{j-1}} \end{array}$$
    * Finally, $U_{i}$ computes any ${\left\{K_{j^{\prime \prime }}\right\}}_{j^{\prime }\leq j^{\prime \prime }\leq j}$ by decrypting ${\left\{E_{h_{2}\left(g^{k_{j}^{j^{\prime \prime }}}\right)}\left(K_{j^{\prime \prime }}\right)\right\}}_{j^{\prime }\leq j^{\prime \prime }\leq j}$.
  - For a revoked user $U_{i}$, $A_{j}^{j^{\prime }}\left(i\right)=0$. Thus, he can not obtain any information about $K_{j}$.
- **GroupMemberAddition**
  When a user, $U_{v}$, joins the group in session *j*, GM randomly selects a unique identity *v* and a session identifier $\varepsilon_{j}$ from $F_{p}$, and distributes a personal key $S_{v}=\varepsilon_{j}\cdot f\left(v\right)$ to him via a secure communication channel. For security, GM starts a new session.
- **GroupMemberRevocation**
  When a user, $U_{i}$ joins the group in session $j^{\prime }$ and is revoked in session *j*, GM includes (x−i) in $A_{j}^{j^{\prime }}\left(x\right)$ and starts a new session.

**Remark 2.**  *In this scheme, the algorithm “Self Healing" is contained in algorithm “Session key recovery". That is, for a legitimate user* $U_{i}$, *by running the algorithm “Session key recovery", he can compute the current session key and recover lost session keys from current broadcast, that is, a self-healing property.*

**Remark 3.**  *For a certain session, say* $j^{\prime \prime }$, *if there are not users joining group in session* $j^{\prime \prime }$, *then* $R_{j}^{j^{\prime \prime }}=\emptyset$, *thus we do not need to compute* $g^{\Phi_{j}^{j^{\prime \prime }}\left(x\right)}$ *and* $\left\{E_{h_{2}\left(g^{k_{j}^{j^{\prime \prime }}}\right)}\left(r_{j^{\prime \prime }}\right)\right\}$. *Suppose the number of the sessions in which the new users join is v, Then, the number of the polynomials* ${\left\{g^{\Phi_{j}^{j^{\prime }}\left(x\right)}\right\}}_{j^{\prime }=1,2,\cdots ,j}$ *and the encrypted message* ${\left\{E_{h_{2}\left(g^{k_{j}^{j^{\prime }}}\right)}\left(r_{j^{\prime }}\right)\right\}}_{j^{\prime }=1,2,\cdots ,j-1}$ *in the broadcast messages* $B_{j}$ *are v, respectively. Note that* v≤j. *Especially, if v is much smaller than m, the communication overhead would be reduced remarkably.*

## 5. Security Analysis and Performance Comparison

In this section, the security and the performance of new E-SGKD scheme will be analyzed.

### 5.1. Security Analysis

We present four theorems and proofs for the new E-SGKD scheme, which demonstrate that the new E-SGKD scheme has the security properties as defined in security model.

**Theorem 5.**  *The new E-SGKD scheme is a secure SGKD scheme with a self-healing property and* mt-*revocation capability.*

**Proof.**  According the definition in Section 2.3, the new E-SGKD scheme has a self-healing property and mt-revocation capability, because it satisfies the following conditions:

(1) For a legitimate user $U_{i}\in G_{j}^{j^{\prime }}$, he can recover the session key $K_{j}$ by combining $B_{j}$ with his personal secret $S_{i}$ as described in the SessionKeyRecovery procedure.
(2) On one hand, the session key $K_{j}$ has a relationship with the initial value of the *j*-th masking key chain, $k_{j}^{1}$, and $r_{j^{\prime }}\left(1\leq j^{\prime }\leq j\right)$. However, because of the revocation polynomial, it is difficult to compute $k_{j}^{1}$ and $r_{j^{\prime }}\left(1\leq j^{\prime }\leq j\right)$ only using the broadcast messages. Therefore, using the broadcast message $B_{j}$ alone can not obtain any information about $K_{j}$. On the other hand, $K_{j}$ is chosen randomly and is independent of the personal secret so that using the personal secret alone can not obtain any information about $K_{j}$.
  Thus, either the broadcast messages or the personal secrets alone can not obtain any information about $K_{j}$.
(3) We first consider a single user $U_{i}\in R_{j}$. For any revoked user $U_{i}\in R_{j}$, if $U_{i}\in R_{j}^{j^{\prime }}$, $A_{j}^{j^{\prime }}\left(i\right)=0$. Hence, he can not obtain any information about $k_{j}^{j^{\prime }}$. Thus, for any revoked user, he alone can not obtain any information about $K_{j}$. Furthermore, we consider the collusion of the users in $R_{j}$. Because the personal secret of a user has a relationship with a session in which he joins the group, only the users joining the group in the same session can collude together. According to the Lagrange Interpolation method, only at least t+1 users coalesce to recover the corresponding $\varepsilon_{j}\cdot f\left(x\right)$. Since $|R_{j}^{j^{\prime }}|\leq t$, the coalition of users in $R_{j}$ can not obtain $\varepsilon_{j}\cdot f\left(x\right)$. Therefore, $U_{i}\in R_{j}$ can not obtain information about $K_{j}$.
(4) From the SessionKeyRecovery procedure, we learn that a legitimate user can recover the lost session keys from his joined session to the current session, which demonstrates that the new scheme has a self-healing property.

**Theorem 6.**  *The new E-SGKD scheme has* mt-*wise forward secrecy.*

**Proof.**  We first consider a single user $U_{i}\in R_{j}$ who tries to recover the session key $K_{j+1}$. For a revoked user $U_{i}\in R_{j}^{j^{\prime }}$, $A_{j+1}^{j^{\prime }}\left(i\right)=0$. Therefore, $U_{i}$ can not obtain any information about $k_{j+1}^{j^{\prime }}$. Hence, $U_{i}$ can not recover $K_{j+1}$.

Now we consider the collusion of the users in $R_{j}$. As described above, only at least t+1 users who join the group in same session can collude to recover $\varepsilon_{j^{\prime }}\cdot f\left(x\right)$. However, $|R_{j}^{j^{\prime }}|\leq t$ so that $\varepsilon_{j^{\prime }}\cdot f\left(x\right)$ can not be recovered. Thus, $U_{i}$ can not obtain any information about $k_{j+1}^{j^{\prime }}$ and the session key $K_{j+1}$. Therefore, the new E-SGKD scheme achieves mt-wise forward secrecy.

**Theorem 7.**  *The new E-SGKD scheme guarantees* any-*wise backward secrecy.*

**Proof.**  Users in $D_{j}$ have to know at least *t*+1 users’ personal secrets $\varepsilon_{j^{\prime }}\cdot f\left(i\right)\;\left(j^{\prime }\leq j\right)$ which are distributed to those users who join the group in the same session, so that they can recover $\varepsilon_{j^{\prime }}\cdot f\left(x\right)$, and, furthermore, recover the session key $K_{j}$. However, according to the definition of $D_{j}$, users in $D_{j}$ join the group after the session *j*, so they only have $\varepsilon_{j^{\prime \prime }}\cdot f\left(i\right)\left(j^{\prime \prime }\geq j+1\right)$. Thus, no matter how many users in $D_{j}$ coalesce, they do not have enough personal secrets to recover $K_{j}$. Therefore, the new E-SGKD scheme guarantees any-wise backward secrecy.

**Theorem 8.**  *The new E-SGKD scheme has* mt-*collusion attack resistance capability.*

**Proof.**  Suppose $R_{j_{1}}$ consists of all users revoked before and in session $j_{1}$, and $D_{j_{2}}$ includes all users joining the group after session $j_{2}$ ($j_{1}<j_{2}$). Even if users in $R_{j_{1}}$ collude with users in $D_{j_{2}}$, they can not recover $K_{j}$ with the knowledge of $B_{j_{1}}$, $B_{j_{2}}$ and users’ personal secrets.

On one hand, a user $U_{i}\in R_{j_{1}}^{j^{\prime }}\left(j^{\prime }<j_{1}\right)$ only has $\varepsilon_{j^{\prime }}\cdot f\left(i\right)$, and a user $U_{v}\in D_{j_{2}}^{j^{\prime \prime }}\left(j^{\prime \prime }>j_{2}\right)$ only has $\varepsilon_{j^{\prime \prime }}\cdot f\left(v\right)$. However, only users joining the group in the same session can collude together and $|R_{j_{1}}^{j^{\prime }}|\leq t$, $|D_{j_{2}}^{\prime \prime }|\leq t$. Even if users in $R_{j_{1}}$ collude with users in $D_{j_{2}}$, they can not obtain enough information to recover $\varepsilon_{j^{\prime }}\cdot f\left(x\right)$ and $\varepsilon_{j^{\prime \prime }}\cdot f\left(x\right)$.

On the other hand, from Theorems 2 and 3, we learn that either the collusion of users in $R_{j_{1}}$ or the collusion of users in $D_{j_{2}}$ alone can not recover $K_{j}$.

Therefore, the new E-SGKD scheme has mt-collusion attack resistance capability.

### 5.2. Performance Comparison

In this subsection, we compare the basic E-SGKD scheme and the new E-SGKD scheme with the previous E-SGKD schemes from the security performance and the efficiency performance. Except for the published E-SGKD schemes, only Liu *et al.*’s Scheme 3 [18] and Hong *et al.*’s Scheme 2 [22] can be converted to the E-SGKD schemes. Here, “Liu *et al.*’s improved scheme" means the E-SGKD scheme constructed from Liu *et al.*’s Scheme 3 using the similar method in Section 3. In general, let *p* be a 128-bit integer and *q* be a 512-bit integer.

#### 5.2.1. The Security Performance

From Table 2, it is easy to find that Construction 5 [1], Scheme 4 [2], "Liu *et al.*’s improved scheme" and our basic scheme do not satisfy the backward secrecy and are not resistant to the collusion attack. The Schemes [14,15] and our new scheme have all of the basic security properties, *i.e.*, forward secrecy, backward secrecy and resistance to collusion attack capability.

Additionally, our new scheme allows more users to be revoked and more users to be colluded together compared with the E-SGKD schemes [14,15]. Note that our new scheme has the capability of resisting mt-wise collusion attack and mt-wise forward secrecy, when there are users joining the group in every session. Our new scheme has the capability of resisting vt-wise collusion attack and vt-wise forward secrecy, when the number of the sessions in which there are users joining group is v(v<m). Specially speaking, the collusion users are less than *t* for each session. Thus, the total number of the collusion users is less than vt.

#### 5.2.2. The Storage Overhead

Now, we focus on the efficiency performance, including the storage overhead and the communication overhead. From Table 3, it is obvious that only scheme [15], our basic scheme and our new scheme have the constant storage overhead, *i.e.*, $\log_{2}p$, which is optimal compared with other E-SGKD schemes.

#### 5.2.3. The Communication Overhead

From Table 3, we can find that the communication overhead of Blundo *et al.*’s Scheme 4 [2], $\left[tj+m+j\right]\log_{2}q+tj\log_{2}p$, is smaller than that of Construction 5 in [1] and Liu *et al.*’s improved scheme. In addition, the communication overhead of scheme [15], $\left(d+1\right)[\left(t+2\right)\log_{2}q+\left(t+1\right)\log_{2}p]$ is smaller than that of scheme [14], $\left(d+1\right)[\left(t+2\right)\log_{2}q+\left(3t+1\right)\log_{2}p]$, where *d* is the size of sliding windows. Therefore, we mainly compare our basic E-SGKD scheme and our new E-SGKD scheme with Blundo *et al.*’s scheme 4 and scheme [15].

In the basic scheme, the broadcast message $B_{j}$ in session *j* includes $R_{i}$, $\left\{g^{P_{j^{\prime }}\left(x\right)}\right\}$, $\left\{g^{v_{j^{\prime }}}\right\}$, where $j^{\prime }=1,2,\cdots ,j$. Because the users’ identities can be chosen from a small finite, the communication overhead of $R_{j}$ can be neglected. Thus, the communication overhead is $\left(t+2\right)j\log_{2}q$, which is obviously less than the communication overhead of Blundo *et al.*’s Scheme 4, based on the fact that j≤m. Similarly, in the new scheme, the broadcast message $B_{j}$ in session *j* includes $R_{j}$, $R_{j}^{\prime }$, $\left\{g^{\Phi_{j}^{j^{\prime }}\left(x\right)}\right\}$, $\left\{E_{h_{2}\left(g^{k_{j}^{j^{\prime }}}\right)}\left(r_{j^{\prime }}\right)\right\}$, $\left\{E_{h_{2}\left(g^{k_{j}^{j^{\prime }}}\right)}\left(K_{j^{\prime }}\right)\right\}$ and $g^{v_{j}}$, where $j^{\prime }\in \left\{1,2,\cdots ,j\right\}$. Note that the communication overhead of $R_{j}$, $R_{j}^{\prime }$ can be neglected. Thus, the communication overhead is $\left[\left(t+1\right)v+1\right]\log_{2}q+\left(v+j\right)\log_{2}p$, where *v* is the number of the sessions in which the new users join. Let $B_{1}=\left(t+2\right)j\log_{2}q$, $B_{2}=\left[\left(t+1\right)v+1\right]\log_{2}q+\left(v+j\right)\log_{2}p$ and v=j which means all sessions have new users join in, and let *p* be a 128-bit integer and *q* be a 512-bit integer. Then, we have:

$$\begin{array}{cc} & B_{1}-B_{2}\\ = & \left(t+2\right)j\log_{2}q-\left\{\left[\left(t+1\right)v+1\right]\log_{2}q+\left(v+j\right)\log_{2}p\right\}\\ = & \left(tj+2j-tj-j-1\right)\log_{2}q-2j\log_{2}p\\ = & \left(j-1\right)\log_{2}q-2j\log_{2}p\\ = & 512(j-1)-128\times 2j\\ = & 256(j-2) \end{array}$$

It is obvious that the communication overhead in our new scheme is lower than the communication overhead of our basic scheme as long as j>2.

Now, we analyze the relationship between the maximum size of the broadcast message and the degree *t* for different E-SGKD schemes. Suppose [x] denotes that *x* rounds down to the nearest whole unit. Let m=30, and *t* varies from 10 to 30.

**Figure 1:**
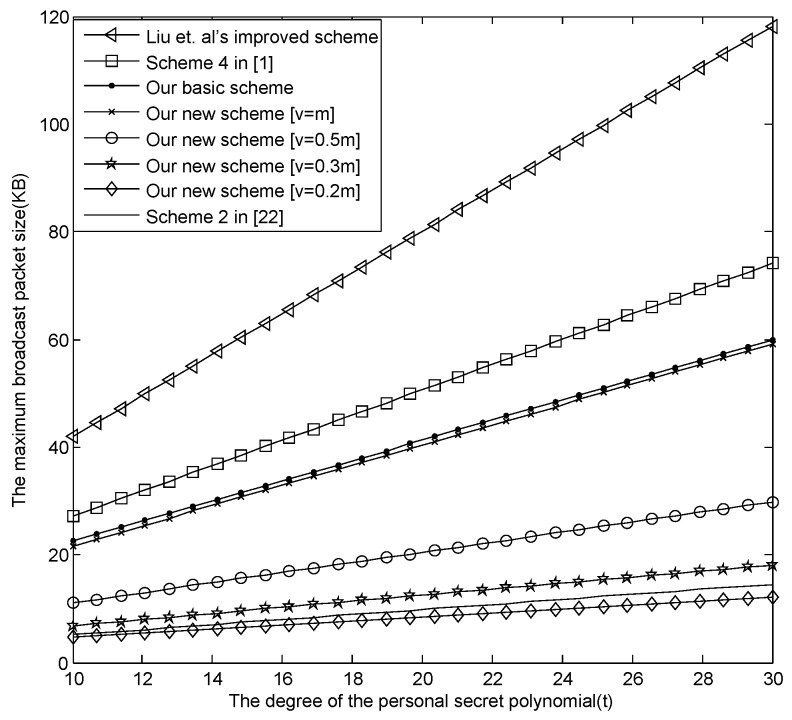
The first comparison of the broadcast message.

**Figure 2:**
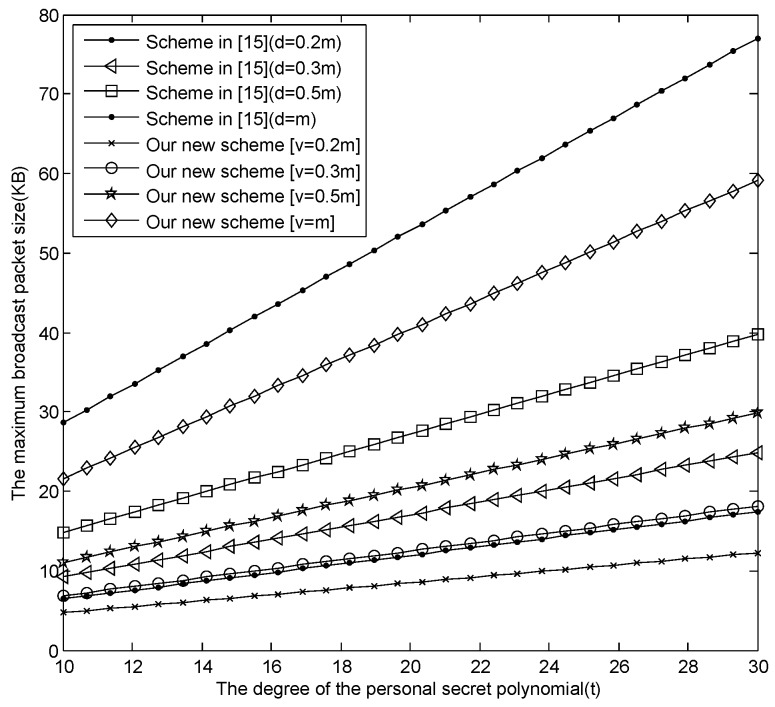
The second comparison of the broadcast message.

(1) The comparison among our basic E-SGKD scheme, our new E-SGKD scheme and Blundo *et al.*’s Scheme 4.
  Figure 1 describes the maximum size of the broadcast message changing with *t*, where v=[0.2m], [0.3m], [0.5m] and [v=m] are discussed, respectively. From Figure 1, when m=30,t=30, the maximum size of the broadcast message in our new E-SGKD scheme is nearly 12.25, 18.1094, 29.8281 and 59.125 KB with *v* = 6, 9, 15 and 30, while the maximum size of the broadcast message in Liu *et al.*’s improved scheme, Blundo *et al.*’s Scheme 4 and our basic E-SGKD scheme is nearly 118.1875, 74.0625 and 60 KB, respectively. Thus, our new E-SGKD scheme has the best performance, especially when the number of the joining sessions *v* is small since the size of the broadcast messages reduce with the value of *v* reduction. Note that when v=[0.2m], the maximum size of the broadcast message in our new E-SGKD scheme is nearly 12.25 KB, which is even smaller than that (say 14.5313 KB) of Hong *et al.*’s Scheme 2 [22], which is the most efficient known P-SGKD scheme.
(2) The comparison between our new E-SGKD scheme and scheme [15]
  The communication overhead in scheme [15] is $\left(d+1\right)[\left(t+2\right)\log_{2}q+\left(t+1\right)\log_{2}p]$, which has a relationship with the size of the sliding windows. In general, we assume that the size of sliding windows (say *d*) is equal to the number of sessions (say *v*), in which there are users joining the group. Figure 2 describes the maximum size of the broadcast message changing with *t*, where v=d=[0.2m], [0.3m], [0.5m] and [v=m] are discussed, respectively. From Figure 2, when m=30,t=30, the maximum size of the broadcast message in our new E-SGKD scheme is nearly 12.25, 18.1094, 29.8281 and 59.125 KB with *v* = 6, 9, 15 and 30, while the maximum size of the broadcast message in scheme [15] is nearly 17.39, 24.84, 39.75 and 77.01 KB, respectively. Thus, our new E-SGKD scheme has lower communication overhead, when v=d.

To sum up, our new E-SGKD scheme has a smaller broadcast size compared with the E-SGKD Schemes [1], [2] and "Liu *et al.*’s improved scheme". In addition, our new E-SGKD Scheme has a smaller broadcast size than the E-SGKD Scheme [15] when v=d. Hence, our new E-SGKD Scheme is efficient in terms of the communication overhead.

### 5.3. Practicality

Many specific issues should be taken into consideration when an SGKD Scheme is applied to real-word scenarios. First, the SGKD Sscheme should work well and efficiently complete the task of the key distribution in the specific scenarios. Second, the system parameters for these scenarios should be determined so that the SGKD Scheme and corresponding parameters can work efficiently.

As we know, for most of the SGKD schemes, the largest broadcast packet is supposed to be 64 KB, so the system parameters should be selected according to the principle of reducing the largest broadcast packet. The largest broadcast packet in the E-SGKD scheme is mainly determined by *p*, *q*, *m* and *t*. In general, suppose *p* is a 128-bit integer and *q* is a 512-bit integer. Then, we simulate the E-SGKD schemes that are applied in the wireless network in which the broadcast packet is 64 KB. The simulation results will contribute in analyzing how to select parameters.

**Figure 3:**
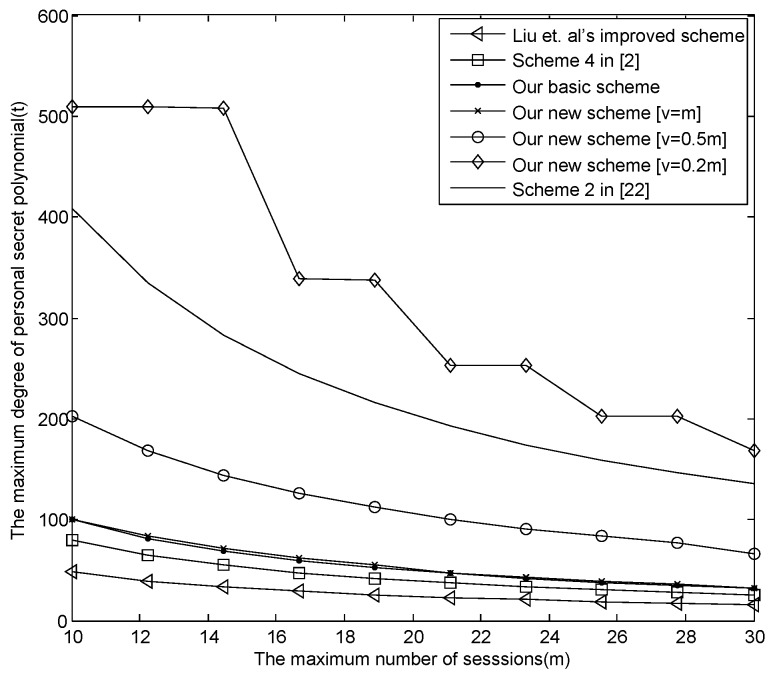
The trade-off between *m* and *t* as the size of the broadcast message is 64 KB.

**Figure 4:**
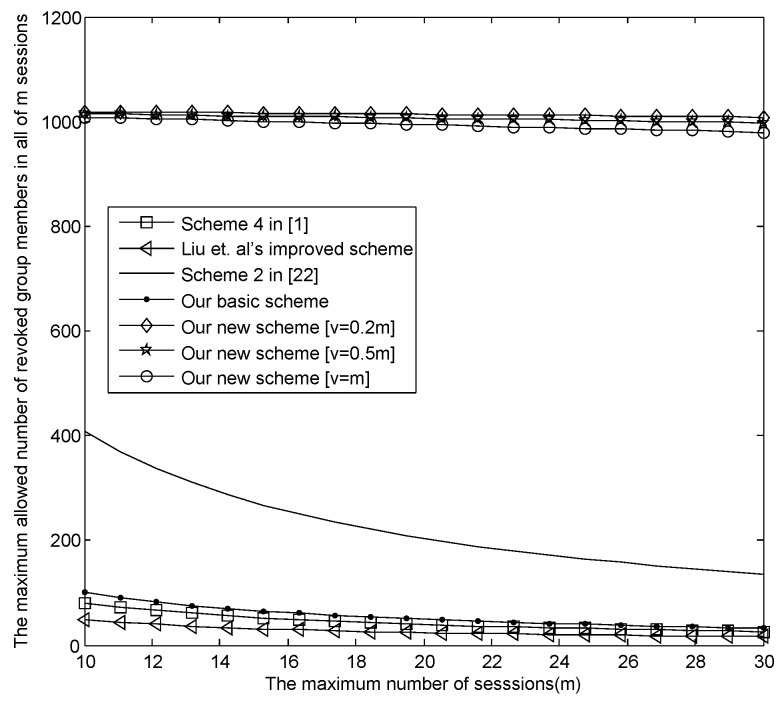
The trade-off between *m* and $|R_{m}{|}_{max}$ as the size of the broadcast message is 64 KB.

(1) The relationship between *m* and *t*.
  Figure 3 describes the relationship between *m* and *t* in the known E-SGKD schemes, our basic E-SGKD scheme and our new E-SGKD scheme, when the largest broadcast packet is constrained to 64 KB. From Figure 3, when m=10, the largest degree of personal secret polynomial *t* in our basic E-SGKD scheme and our new E-SGKD scheme with v=m are closed, *i.e.*, 100 and 94, respectively, while *t* reaches 46 and 80 in “Liu *et al.*’s improved scheme" and Blundo *et al.*’s scheme [2]. When m=30, the values of *t* in our basic E-SGKD scheme and our new E-SGKD scheme with v=m are the same, *i.e.*, 32, while the values of *t* in “Liu *et al.*’s improved scheme" and Blundo *et al.*’s Scheme [2] are 15 and 25, respectively.
  It is obvious that the range of *m* and *t* in our new scheme is larger than the range in other E-SGKD schemes when v<m. For example, when v=[0.5m] and v=[0.2m], where m=10, the values of *t* in our new E-SGKD scheme are increased remarkably, say 202 and 509, respectively. Note that, when v=[0.2m], the value of *t* even is larger than the value of *t* (say 410) in the most efficient known P-SGKD scheme, *i.e*, Hong *et al.*’s Scheme 2 [22]. Thus, our new E-SGKD scheme has the best performance.

- The relationship between *m* and the maximum revoked users in all sessions $|R_{m}{|}_{max}$.
  Figure 4 presents the relationship between *m* and the maximum revoked users in all sessions $|R_{m}{|}_{max}$, where v=[0.2m], [0.3m], [0.5m] and [v=m], respectively. From Figure 4, when m=30, $|R_{m}{|}_{max}$ in our new E-SGKD scheme is nearly 1000 with *v* = 6, 9, 15 and 30, while $|R_{m}{|}_{max}$ in Liu *et al.*’s improved scheme, Blundo *et al.*’s Scheme 4 and our basic E-SGKD scheme is nearly 15, 25 and 32, respectively. Additionally, we can find that $|R_{m}{|}_{max}$ in the most efficient P-SGKD scheme, *i.e.*, Hong *et al.*’s Scheme 2 [22], is 135. Thus, our new E-SGKD scheme allows more revoked users than all other known E-SGKD schemes and P-SGKD schemes, *i.e.*, the new scheme can resist more users’ collusion attacks.

## 6. Practicality in ZigBee Network

In this section, we mainly discuss how to apply our new E-SGKD scheme to special kinds of resource-constrained wireless sensor networks, *i.e.*, ZigBee networks. For the resource-constrained wireless networks, resources including the users’ storage and communication bandwidth is limited.

ZigBee protocol is designed for low-data-rate wireless networks and is very suitable for low-rate, low-cost and low-energy-consumption networks. As we know, for the ZigBee protocol [38,39], the maximum size of the Mac layer data is from 89 to 119 bytes. When the the maximum size of the Mac layer data is 89 bytes, if the data of the application layer are more than 89 bytes, the data will be divided into blocks. Assume that the maximum size of the broadcast message are 4 KB. Then, the broadcast message will be divided into 46 small packets with 88 bytes/packet. Without loss of generality, suppose packets are lost randomly and independently. When the packet loss rate is 1%, only 37.01% of packets reach their destination. When the packet loss rate is 5%, only 9.45% of packets reach their destination, *i.e.*, only one packet in ten reaches its destination and is received by a group member. Hence, suppose *m* is at least 10.

**Figure 5:**
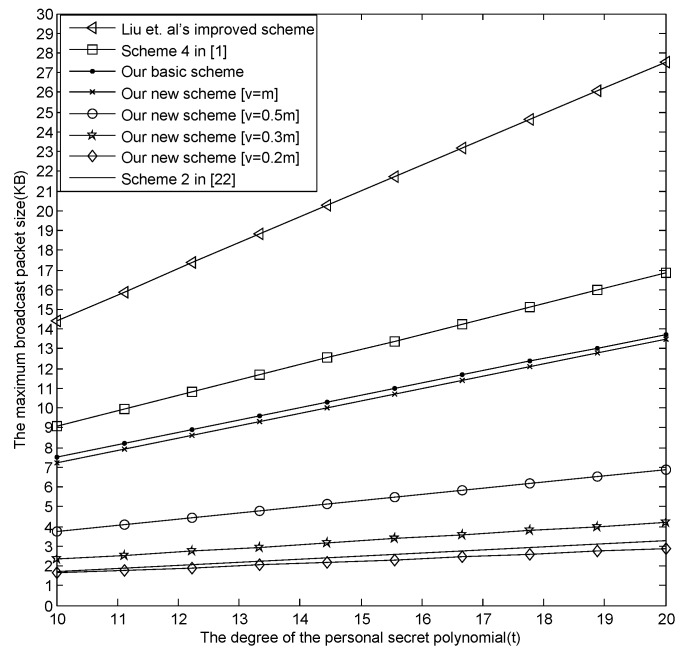
The comparison of the broadcast message when *m* is 10.

Under the above assumption, *i.e.*, *m* equals 10 and the maximum size of the broadcast message is 4 KB, we now check if the known E-SGKD schemes are suitable for ZigBee wireless networks or not. From Figure 5, we can find that when *t* = 10, the size of the broadcast message is more than 4 KB in the most efficient E-SGKD scheme, *i.e.*, our new scheme with [v=m], and is less than 4 KB in our new E-SGKD scheme with v=[0.2m], [0.3m] and [v=m] and Hong *et al.*’s efficient P-SGKD Scheme 2. Meanwhile, the size of the broadcast message increases as *t* increases. When *t* reaches 20, the size of the broadcast message is more than 7 KB in our new scheme with [v=0.5m], while is still less than 4 KB in our new E-SGKD scheme with v=[0.2m], [0.3m] and Hong *et al.*’s efficient P-SGKD scheme. Note that when v=[0.2m], the communication overhead is even lower than the most efficient P-SGKD scheme.

Thus, we can conclude that all of the known E-SGKD schemes can not be applied to the ZigBee network, and only our new E-SGKD scheme with v=[0.2m], [0.3m] is suitable for the ZigBee network, since it also has optimal storage overhead.

Now, we discuss how to select the system parameters when applying our new E-SGKD scheme to the ZigBee-based wireless network. The simulation results will contribute to analyzing how to select parameters. Suppose the largest broadcast packet in ZigBee-based wireless networks is 4 KB. As discussed above, we only analyze our new E-SGKD scheme with v=[0.2m], [0.3m].

**Figure 6:**
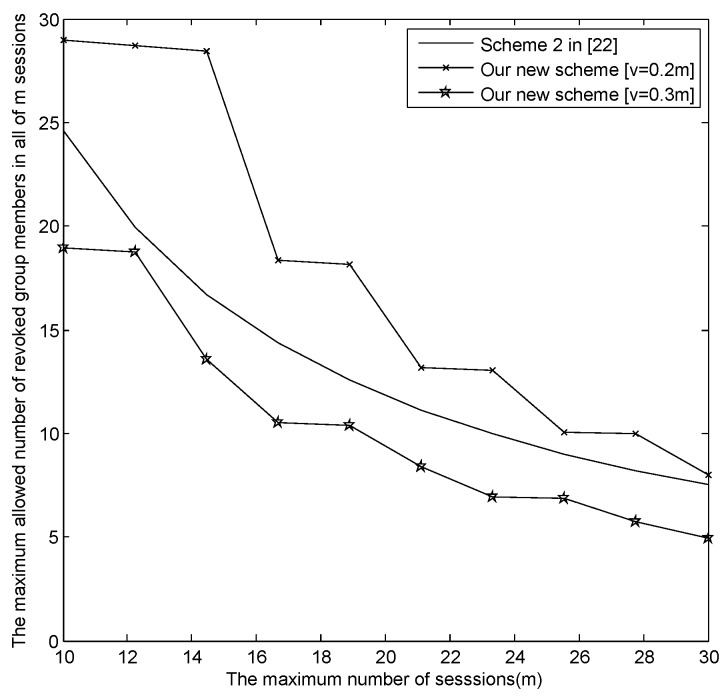
The trade-off between *m* and *t* as the size of the broadcast message is 4 KB.

From Figure 6, the relationship between *m* and *t* in our new E-SGKD scheme with v=[0.2m], [0.3m] and Hong *et al.*’s efficient P-SGKD Scheme 2 [22] is described when the largest broadcast message is constrained to 4 KB. It is obvious that the range of *m* and *t* in our new E-SGKD scheme with v=[0.2m] is the largest among three schemes. Thus, if the number of the sessions in which the new users join, *i.e.*, *v*, is more smaller, *m* and *t* can be bigger. For example, if v=[0.2m], then m=10 and t=29, or m=15 and t=25, or m=20 and t=15, or m=28 and t=10.

**Figure 7:**
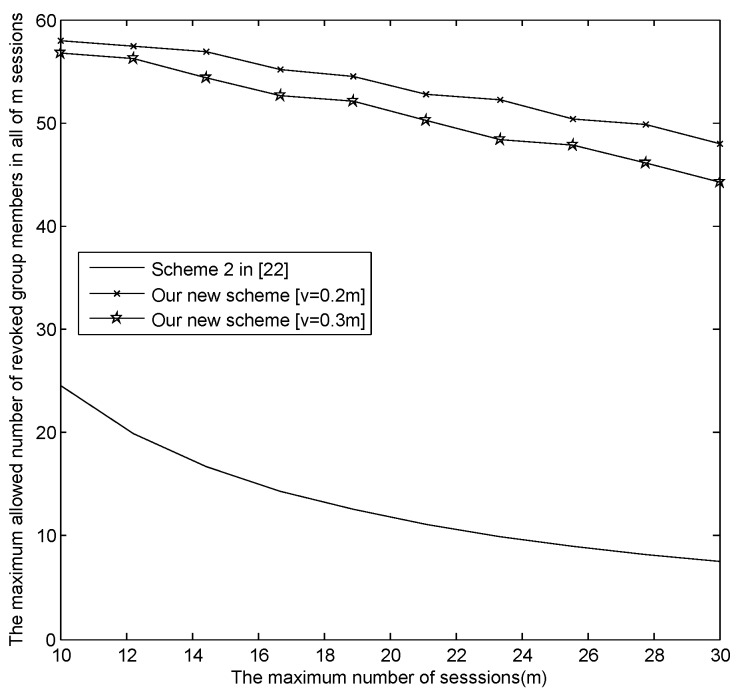
The trade-off between *m* and $|R_{m}{|}_{max}$ as the size of the broadcast message is 4 KB.

Figure 7 presents the relationship between *m* and the maximum revoked users in all sessions $|R_{m}{|}_{max}$, which demonstrates that our new E-SGKD scheme with v=[0.2m] and [0.3m] allows more revoked users than Hong *et al.*’s efficient P-SGKD scheme 2. For example, when m=10, t=29 and 19, with v=2 and 3, the maximum allowed revoked members in all *m* sessions are 58 and 57, while the allowed group members in all *m* sessions in Hong *et al.*’s efficient P-SGKD scheme 2 [22] is only 24. Under the same conditions, when m=30, t=8 and 4, with v=6 and 9, the maximum allowed revoked members in all *m* sessions are 48 and 36, while the allowed group members in all *m* sessions in Hong *et al.*’s efficient P-SGKD Scheme 2 [22] is only eight.

In conclusion, if the new users do not join the group frequently, the number of the sessions in which new users join is small. In this case, our new scheme is efficient in terms of the storage overhead and the communication overhead. When v=[0.2m], the overall efficiency is higher than the most efficient known P-SGKD.

## 7. Application to Supervisory Control And Data Acquisition (SCADA) in Smart Grid

**Figure 8:**
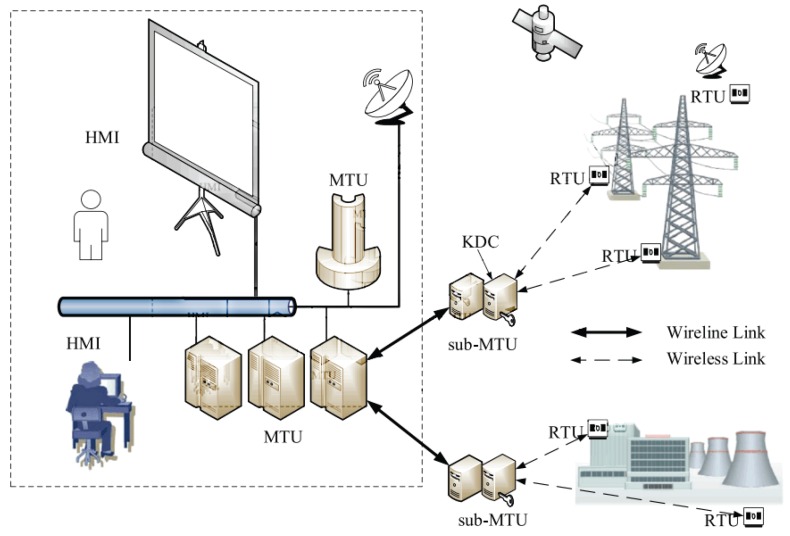
A simple supervisory control and data acquisition system architecture.

Smart grids are becoming more and more important in modern society. SCADA systems are applied to monitor and control smart grids. The SCADA system consists of human-machine interface (HMI), master terminal unit (MTU), and remote terminal unit (RTU). The structure of these entities is as described in Figure 8 ([40]). HMI is a human-computer interaction device. MTU is in charge of supervisory control to the RTUs. As shown in Figure 8, the SCADA system consists of one MTU and multiple sub-MTUs, and these MTUs have rich resources such as storage space and computational capability. Thus, the public key cryptography can be used to protect the security among them. Compared with MTU, the resources of RTUs are limited. In addition, the RTUs are often located in remote places and the security can not be guaranteed.

In SCADA systems, the sensitive data will be transmitted among different parts of the power grid. Key management mechanisms can be used to protect the security of the data. Due to a self-healing property and low storage overhead requirements, our proposed E-SGKD scheme is suitable for achieving the key distribution and resolving the transmission availability and security in resource-constrained SCADA systems, where the sub-MTUs are as the GM and RTUs are as group manager nodes, which can efficiently achieve the key distribution and updating in SCADA systems.

## 8. Conclusions

In this paper, we proposed two E-SGKD schemes. The basic E-SGKD scheme was constructed from a known polynomial-based SGKD, and it has offered the optimal storage overhead while not having backward secrecy. The new E-SGKD scheme was constructed from the basic E-SGKD scheme. To consider the communication overhead and the backward secrecy, a novel approach is introduced for message broadcasting, which makes the new E-SGKD scheme obtain all basic security properties. Compared with known E-SGKD schemes, our new scheme has optimal storage overhead and low communication overhead. We discussed how to select the parameters and simulated it in the ZigBee network. Finally, we introduce the application of our proposed E-SGKD scheme to SCADA systems in the smart grid.

## Figures and Tables

**Table 1:** Notations.

$U_{i}$	the *i*-th user node
*m*	the maximum sessions
*t*	the maximum revoked users
*v*	the number of the sessions in which there are users joined the group (1≤v≤j)
$F_{p}$	a finite field of order *p*, where *p* is a prime
$F_{q}^{*}$	a multiplicative group of finite of order *q*
*g*	a generater of $F_{q}^{*}$
S(i)	$U_{i}$’s personal secret
$E_{k}\left(\cdot \right)/D_{k}\left(\cdot \right)$	symmetric encryption/decryption function
$B_{j}$	the *j*-th key updating broadcast message
$h_{1}\left(\cdot \right),h_{2}\left(\cdot \right)$	one-way hash function
$\varepsilon_{j}$	the unique session identifier, chosen at random by GM for users who joined the group in session *j*, $\varepsilon_{j}\in F_{q}$ and $\varepsilon_{j_{1}}\neq \varepsilon_{j_{2}}$ for $j_{1}\neq j_{2}$
$k_{j}^{1}$	the initial value of *j*-th key chain chosen at random by GM for session *j*, $k_{j}^{1}\in F_{q}$, and $k_{j_{1}}^{1}\neq k_{j_{2}}^{1}$ for $j_{1}\neq j_{2}$
$k_{j}^{j^{\prime }}$	the $j^{\prime }$-th key in the *j*-th key chain
$R_{j}^{j^{\prime }}$	the set of users joining group in session $j^{\prime }$ and revoked before or in session *j* ($j^{\prime }\leq j$)
$|R_{j}^{j^{\prime }}|$	the number of users in $R_{j}^{j^{\prime }}$, and $|R_{j}^{j^{\prime }}|\leq t$
$R_{j}$	the set of users who are revoked before and in session *j*, and $R_{j}=\left\{R_{j}^{1},\cdots ,R_{j}^{j}\right\}$
$|R_{j}|$	the number of users in $R_{j}$
$G_{j}^{j^{\prime }}$	the set of group members joining the group in session *j* and still legitimate in session *j* ($j^{\prime }\leq j$)
$|G_{j}^{j^{\prime }}|$	the number of users in $G_{j}^{j^{\prime }}$
$G_{j}$	the set of legitimate group user in session *j*, and $G_{j}=\left\{G_{j}^{1},\cdots ,G_{j}^{j}\right\}$
$|G_{j}|$	the number of users in $G_{j}$

**Table 2:** Comparison of security properties.

Scheme	Revocation Limit	Forward Secrecy	Backward Secrecy	Collusion Resistance	The Maximum Number of Collusion Attack Resistance
Construction 5 in [1]	*t*	Yes/*t*	No	No	0
Scheme 4 in [2]	*t*	Yes/*t*	No	No	0
Liu *et al.*’s improved scheme	*t*	Yes/*t*	No	No	0
Scheme in [14]	*t*	Yes/*t*	Yes/*t*	Yes	*t*
Scheme in [15]	*t*	Yes/*t*	Yes/*t*	Yes	*t*
Our basic scheme	*t*	Yes/*t*	No	No	0
Our new scheme	mt	Yes/mt	Yes/any	Yes	mt

**Table 3:** Comparison of storage overhead.

Scheme	Storage Overhead	Communication Overhead
Construction 5 in [1]	$\left(m^{2}+2\right)\log_{2}p$	$\left(mt^{2}+2mt+m\right)\log_{2}q+t\log_{2}p$
Scheme 4 in [2]	$m\log_{2}p$	$\left(tj+m+j\right)\log_{2}q+tj\log_{2}p$
Liu *et al.*’s improved scheme	$2m\log_{2}p$	$\left[\left(m+j+1\right)t+\left(2m+1\right)\right]\log_{2}q$
Scheme in [14]	(m−j+2)$\log_{2}p$	$\left(d+1\right)[\left(t+2\right)\log_{2}q+\left(3t+1\right)\log_{2}p]$
Scheme in [15]	$\log_{2}p$	$\left(d+1\right)[\left(t+2\right)\log_{2}q+\left(t+1\right)\log_{2}p]$
Our basic scheme	$\log_{2}p$	$\left(t+2\right)j\log_{2}q$
Our new scheme	$\log_{2}p$	$\left[\left(t+1\right)v+1\right]\log_{2}q+\left(v+j\right)\log_{2}p$
